# Project SELfit: training socio-emotional skills in a school environment in Porto

**DOI:** 10.1093/eurpub/ckac131.493

**Published:** 2022-10-25

**Authors:** F Malcata, A Rodrigues, A Machado, MT Saraiva, D Antunes

**Affiliations:** 1 Public Health Unit, ACES Porto Ocidental, Porto, Portugal; 2 Catholic University of Portugal, Braga Region Centre, Braga, Portugal

## Abstract

**Issue/Problem:**

Mental disorders are the main cause of years lived with disability (YLD) in 5-14-year-old people around the world, in Western Europe, and particularly in Portugal. Young people who experience anxiety during childhood have a 3,5-fold likelihood of suffering from depression or anxiety during adulthood. COVID-19 has aggravated this situation, namely regarding socio-emotional competencies.

**Description of the problem:**

SELfit, a project based on the Socio-emotional learning (SEL) methodology, aims promoting mental health, by training teachers and community nurses to develop socio-emotional skills in students from primary schools. The project includes a theoretical and a practical/training session, and monthly supervision sessions by a team that includes a psychologist, a public health doctor and public health nurses. The period of implementation is between February to July 2022.

**Results:**

A total of 8 community nurses and 13 teachers from 3 primary schools in Porto enrolled in this project, which corresponds to a total around 272 students from 5 to 8-year-old. Knowledge of nurses and teachers regarding mental health literacy, and social emotional learning was assessed, before and after the theoretical session. The mean percentage of correct answers before was 49% (54% regarding nurses and 45% teachers) compared to 84% (92% regarding nurses and 78% teachers) after. Concerning socio-emotional skills of the primary school students, possible improvement will be evaluated at the end of the project (July 2022).

**Lessons:**

Mental health literacy and social emotional learning knowledge increased 35%, which highlights the importance of these theoretical sessions promoting mental health literacy. By the end of this project, it is expected to exist an improvement on socio-emotional skills of the primary school students. This is a very important project, expected to be implement and replicated in other schools in Porto and in Portugal.

**Key messages:**

• Mental disorders are the main cause of years lost due to disability (YLD) in primary school students in Portugal; hence, it was chosen for this mental health promotion project.

• This is a pioneer project in Porto, involving both community nurses and primary teachers, in which all are involved in training and promoting social-emotional learning in a school context.

